# Physicochemical, structural, and adsorption characteristics of DMSPS-*co*-DVB nanopolymers

**DOI:** 10.3389/fchem.2023.1176718

**Published:** 2023-06-28

**Authors:** Alicja Bosacka, Malgorzata Zienkiewicz-Strzalka, Anna Derylo-Marczewska, Agnieszka Chrzanowska, Magdalena Blachnio, Beata Podkoscielna

**Affiliations:** ^1^ Department of Fundamental Technologies, Faculty of Production Engineering, University of Life Sciences, Lublin, Poland; ^2^ Department of Physical Chemistry, Faculty of Chemistry, Institute of Chemical Sciences, Maria Curie-Sklodowska University, Lublin, Poland; ^3^ Department of Polymer Chemistry, Faculty of Chemistry, Institute of Chemical Sciences, Maria Curie Skłodowska University, Lublin, Poland

**Keywords:** polymers, microspheres, surface properties, aniline adsorption, nanomaterials

## Abstract

The aim of this work is the synthesis and characterization of the series of S,S′-thiodi-4,1-phenylene bis(thio-methacrylate)-*co*-divinylbenzene (DMSPS-*co*-DVB) nanomaterials. The series of new nanopolymers including three mixed systems with different ratios of DMSPS and DVB components, DMSPS-*co*-DVB = 1:1, DMSPS-*co*-DVB = 1:2, and DMSPS-*co*-DVB = 1:3, was synthesized in the polymerization reaction. The research task is to investigate the influence of the reaction mixture composition on morphological, textural, and structural properties of final nanosystems including size, shape, and agglomeration effect. The advanced biphasic nanomaterials enriched with thiol groups were successfully synthesized as potential sorbents for binding organic substances, heavy metals, or biomolecules. To determine the impact of the DMSPS monomer on the final properties of DMSPS-*co*-DVB nanocomposites, several techniques were applied to reveal the nano-dimensional structure (SAXS), texture (low-temperature nitrogen sorption), general morphology (SEM), acid–base properties (potentiometric titration), and surface chemistry and phase bonding effectiveness (FTIR/ATR spectroscopy). Finally, kinetic studies of aniline sorption on polymeric materials were performed.

## 1 Introduction

Nanocomposites are currently attracting a lot of attention as they represent a significant class of high-performance materials (Sun et al., 2016; Cao et al., 2023; Hu et al., 2023). Among them, the polymer nanocomposites seem to be very attractive due to their specific characteristics and wide applicability (Kovačič and Krajnc, 2009; Warwar Damouny and Silverstein, 2016; Golub and Krajnc, 2020; Vásquez et al., 2021). The most significant properties of the polymeric nanocomposites (textural, morphological, structural, mechanical, and thermal properties, as well as their stability and affinity to various substances) can be widely controlled by the synthesis process with regard to their further applicability (Hasegawa et al., 2011; Lin et al., 2013; Chen et al., 2017). Among them, polymers with a porous structure and spherical morphology, which are sometimes referred to as “smart particles,” are quite important due to their high loading/release behavior toward various substances (Kim et al., 2006; Wang et al., 2006; Li et al., 2010). The polymeric materials reveal potential as three-dimensional scaffolds in biomedical applications (Taek et al., 2006; Wang et al., 2009) and chemical engineering (Bernards and Desai, 2010; Shim and Kwon, 2012; Chen et al., 2020; Jiang et al., 2022). For example, highly anisotropic nanoparticles with controlled dimensions based on a morphological transformation process of the adenine- and thymine-functionalized monomers can be obtained (Hua et al., 2019). Several of the most important advantages of such porous polymers include the possibility of fabrication of materials with various sizes of pores, densities, and morphologies. These factors play a crucial role in determining the most important properties, taking into account their further applicability (Kim et al., 2018). Here, the authors emphasize further advantages of polymer nanostructures, including relatively low-cost and scalable syntheses, the possibility of tuning chemical composition, and control of material characteristics. Several reports exist on the fabrication of polymeric particles with non-spherical geometries with several distinct shapes (Manoharan et al., 2003; Rolland et al., 2005). Elsewhere, the construction of precise soft matter nanostructures as centipede-like polymer nanowires with uniform topographical features and internal compartmentalization was proposed (Pelras et al., 2018). The 1D polymer nanomaterials as cylindrical polymer brushes with a high level of control over the backbone and side chain lengths, and their distribution (Verduzco et al., 2015), multicompartment particles with collapsed and patchy shells (Synatschke et al., 2013), and many others were also proposed (Cui et al., 2007; Löbling et al., 2014).

The morphological and topographical features of NPs are very important from a practical point of view. It should be emphasized that the properties of nanomaterials are strongly correlated to grain shape which is attained during the growth and formation of nanomaterials through a self-assembling (or other mechanisms) process influenced by the interplay of various molecular interactions. The following features are used to describe the morphology: size, shape, dispersity, localization, agglomeration/aggregation, surface morphology, surface area, and porosity. Not without significance is the functionality of the surface layers, which plays an important role in the interaction between the components first at the synthesis stage and then at the stage of use in, e.g., sorption processes. Some recent examples include the preparation of hierarchically porous spherical particles with thiol functionality and interconnected cellular morphology in a batch process (Kramer and Krajnc, 2021).

In this work, S,S′-thiodi-4,1-phenylene bis(thio-methacrylate)-*co-*divinylbenzene (DMSPS-*co*-DVB) materials were synthesized by a copolymerization reaction at three different molar ratios 1:1, 1:2, and 1:3. The DMSPS is a representative of methacrylate thiol (a symmetric large organic molecule with sulfur atoms and carboxyl groups), and the DVB is a benzene derivative with two vinyl groups (Supplementary Figure S1). The proposed biphasic nanomaterials show promising properties, especially as adsorbents for the removal of metals and organic compounds from aqueous solutions and as selective materials for solid-phase extraction techniques because of their unique physicochemical properties. To assess the effect of the DMSPS component on the final properties of DMSPS-*co*-DVB nanomaterials, several techniques were applied to indicate the nano-dimensional structure (SAXS), texture (low-temperature isothermal nitrogen sorption), morphology (SEM), acid–base properties (potentiometric titration), surface chemistry, and phase bonding efficiency (FTIR/ATR spectroscopy). In addition, the kinetic studies of aniline (AN) sorption on polymeric adsorbents were performed.

## 2 Experimental

### 2.1 Chemicals and materials

The S,S′-thiodi-4,1-phenylene bis(thio-methacrylate) (DMSPS), α,α′-azoiso-bis-butyronitrile (AIBN), and divinylbenzene (DVB) (62.2% of 1,4-divinylbenzene and 0.2% of 1,2-divinylbenzene and ethylvinylbenzene) were purchased from Merck (Darmstadt, Germany). Hydrochloric acid, sodium hydroxide, and aniline were purchased from Avantor Performance Materials Poland S.A. (Gliwice, Poland).

The chemical structures of DMSPS and DVB monomers used in the polymerization reaction are shown in Supplementary Figure S1. The properties of aniline used as an adsorbate adsorption studies are shown in Supplementary Table S1 (Supplementary Material). The polymerization reaction was carried out according to the procedure proposed by Fila et al. (2018), but different mass ratios of monomers were used: 1:1, 1:2, and 1:3 of DMSPS and DVB, respectively.

### 2.2 Methods

#### 2.2.1 Microscopy and structural analysis

The SEM analysis was performed by using the Quanta^TM^ 3D FEG (FEI Company, Hillsboro, OR, United States) apparatus operating at 5 kV. A high-vacuum (4 × 10^−4^ Pa) mode for imaging the investigated samples was applied. Before measurements, the samples were mounted on aluminum stubs and sputtered with gold.

The SAXS analysis was carried out by using an Empyrean diffractometer (Panalytical) using a CuKα radiation source as SAXS/WAXS in capillary mode configuration. The SAXS configuration includes a 2θ range of 0.1–2° which corresponds to *q* values of 0.0095–0.15 Å^−1^. The length of the scattering vector *q* is defined as *q* = 4πsin*θ*/*λ*, where 2*θ* is the scattering angle and *λ* is the X-ray wavelength (1.5418 Å). During measurements, the device was powered by a 4 kW high-voltage X-ray generator with settings of 40 kV and 40 mA. The incident beam path consisted of a line focus type, W/Si, graded X-ray mirror with an elliptic shape. The primary beam was measured using a beam attenuator Cu 0.2 mm. The measurements were taken using a PIXcel^1D^ detector and receiving slit with 0.05 mm active length. Background scattering was performed by air scattering measure with an empty sample holder. EasySAXS software was applied for SAXS calculations. *Dv(R)* calculations were performed using the indirect Fourier transformation technique with an algorithm based on Tikhonov’s regularization method (Sun et al., 2016). Pair distance distribution function (PDDF) as real space counterparts of the experimental intensity *p(r)* was calculated by the EasySAXS program as an indirect Fourier transform of these data. The Guinier plot as *ln*(*I(q)*) *vs*. *q* ^2^ was used to determine the radius of gyration *R*_g_ from the slope of the plot *− R*_*g*_^*2*^ */3*.

The textural properties of DMSPS-*co*-DVB materials were examined using low-temperature nitrogen adsorption–desorption isotherms at 77 K (ASAP 2020 analyzer, Micromeritics, United States). The values of the parameters characterizing the surface properties of investigated materials were determined: the BET specific surface area (*S*_*BET*_) (assessed from the linear BET plot of adsorption data), the total pore volume (*V*_*t*_) (from the adsorption value at the relative pressure *p/p*_*0*_ ∼ 0.99), and the micropore volume (*V*_*MIC*_) (from the t-plot). The averaged hydraulic pore diameter (calculated according to the equation: *D*_*h*_ = *4V*_*t*_ */S*_*BET*_). Before the measurement, the volatile substances adsorbed on the surface of the material were removed during sample degassing (100°C) for 24 h.

The potentiometric titration was performed using a system equipped with a pH meter (PHM240 Radiometer, Copenhagen, Denmark), an autoburette 765 Dosimat (Metrohm, Herisau, Switzerland), and a thermostat (Ecoline RE 207, Lauda, Germany). The 0.1 mol/L electrolyte (NaCl) with a volume of 30 mL and 0.3 mL of 0.5 mol//L HCl was placed in a quartz vessel. After 1 h of equilibration of the system to achieve a constant temperature and pH value, the polymeric sample of mass of 100 mg was added. The polymeric suspension in the NaCl/HCl solution mixture (25°C, 110 rpm) was gradually titrated with 0.2 mol/L NaOH. Finally, the pH_PZC_ values of the tested samples were determined based on the crossing of electrolyte and suspended samples curves.

Fourier-transformed infrared connected with attenuated total reflection (FT-IR/ATR) analysis was conducted by using an IR spectrometer TENSOR 27 (Brucker, Germany) equipped with the diamond crystal. The spectra were recorded in the spectral range of 400–4000 cm^−1^.

Kinetic measurements were carried out using a UV–Vis spectrophotometer (Cary 100, Varian, Melbourne, Victoria, Australia) with a flow working cell for periodic measurements of a solution concentration in a closed system. The sample of mass of 100 mg was placed in a quartz vessel connected with a stirrer (110 rpm) and thermostatic system (25°C) and filled with 100 mL of aniline solution (C_0_ = 0.205 mmol/L). At definite time intervals, the solution samples were collected in a flow cell, absorption spectra were measured, and the solution was turned back to the reaction vessel. The concentration profiles in the function of time for the experimental systems were calculated from the recorded spectra. The experimental kinetic data were optimized using various theoretical models that are listed in Table 1.

**TABLE 1:** Various kinetic equations applied to optimize the experimental data (Blachnio et al., 2018; Derylo-Marczewska et al., 2019; Blachnio et al., 2020).

Kinetic model	General equation	Half-time expression	Equilibrium uptake; finish time of experiment uptake
First-order equation (FOE)	$\frac{dc}{dt}={-k}_{1}\left(c-c_{eq}\right)$	*t*_*0.5*_ ∼ 1/k_1_	*u*_*eq*_ *= 1-*(*c*_*eq*_ */c*_*o*_)*;u*_*exp_f*_ *= 1-*(*c*_*exp_f*_ */c*_*o*_)
Second-order equation (SOE)	$\frac{dc}{dt}={-k}_{2c}{\left(c-c_{eq}\right)}^{2}$	*t*_*0.5*_ ∼ 1/k_2_	
Fractal-like MOE equation (f-MOE)	$F=\frac{{1-\exp\left(-k_{1}t\right)}^{p}}{{1-f_{2}\exp\left(-k_{1}t\right)}^{p}}$	*t*_*0.5*_ ∼ [ln (2-f_2_)]^1/p^/k_1_	
Fractal-like FOE equation (f-FOE)	$F={1-\exp\left(-k_{1}t\right)}^{p}$	*t*_*0.5*_ ∼ [(ln2)]^1/p^/k_1_	
Fractal-like SOE equation (f-SOE)	$F=\frac{{\left(k_{2}t\right)}^{p}}{{1+\left(k_{2}t\right)}^{p}}$	*t*_*0.5*_ ∼ 1/k_2_	

*ceq* is the equilibrium concentration, *co* is the initial concentration, *c* is the temporary concentration, *cexp_f* is the concertation at the finish time of the experiment*, k* is the kinetic rate coefficient, *t* is time, *F* is the adsorption progress, and *p* is the fractal parameter.

### 2.3 Results and discussion

**FIGURE 1:**
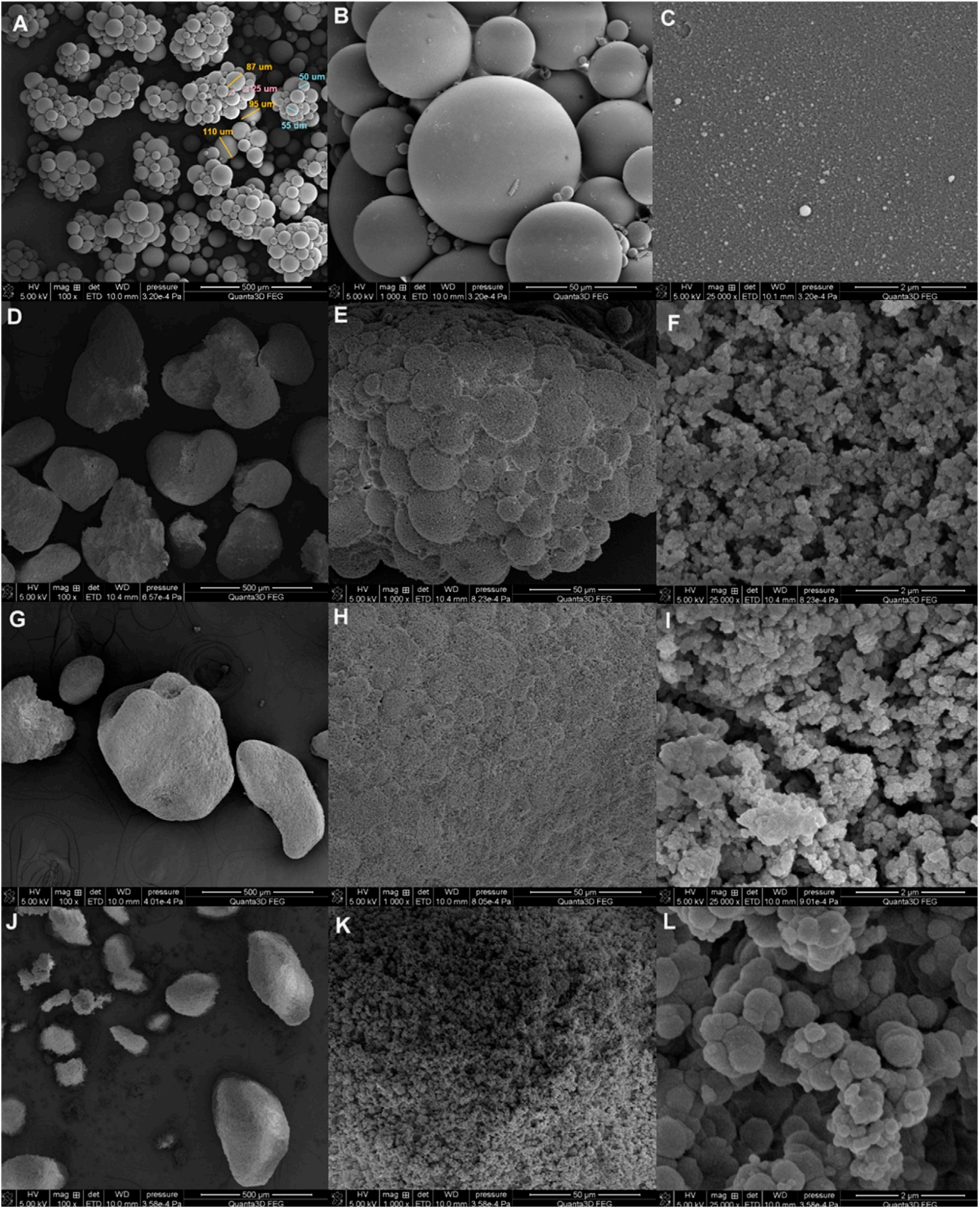
SEM images of DVB **(A–C)** and DMSPS-*co*-DVB samples in different magnifications: DMSPS-*co*-DVB = 1:3 **(D–F)**, DMSPS-*co*-DVB = 1:2 **(G–I)**, and DMSPS-*co*-DVB = 1:1 **(J–L)**.

The morphology and surface nature of the synthesized materials were investigated by direct microscopic observation via scanning electron microscopy (SEM). Furthermore, SAXS and nitrogen adsorption–desorption analysis for describing full nanostructure characteristics were applied. The SEM results in Figure 1 show low- and high-magnification images of the final polymeric nanomaterials. As shown, DVB beads display well-defined spherical shapes and smooth surfaces. Their diameter ranges between 20 um and 120 μm. Small spheres of a few µm between the main beads were also visible. The successive increase in the content of DMSPS changes these originally well-shaped balls. Both their general morphology and surface are modified. Even a small amount of DMSPS (in the DMSPS-*co*-DVB = 1:3 sample) causes the formation of agglomerates of microspheres.

However, the remnant of the original morphology is still observed at higher magnifications (Figure 1E) as the indistinctly outlined spherical elements on the surface. Moreover, the surface of the material is noticeably uneven and not as compact as the DVB sample. Small spherical elements of advanced structures are visible (Figure 1F). The structure of the materials obtained with a greater amount of DMSPS (DMSPS-*co*-DVB = 1:2) further develops according to the aforementioned morphological changes (Figures 1G–I). Here, the morphology disappears almost completely. Similarly, the surface of the material is now very uniform, and the surface spherical system is almost invisible (Figure 1H). Moreover, the dimension of the surface spherical residues decreases. The “fluffy” internal structure becomes reinforced (Figure 1I). The DMSPS-*co*-DVB = 1:1 sample (Figures 1J–L) reveals a completely different morphology than the initial objects. At higher magnifications (Figure 1L), the dependence of larger dimensions of elements building the structure with increasing DMSPS content has been revealed (Figures 1C, E, I, L recorded at the same magnifications). SEM images also suggest that the characteristic feature of the tested materials is their gradual structure; i.e., small elements build larger ones, and these create the next structural step.

**FIGURE 2:**
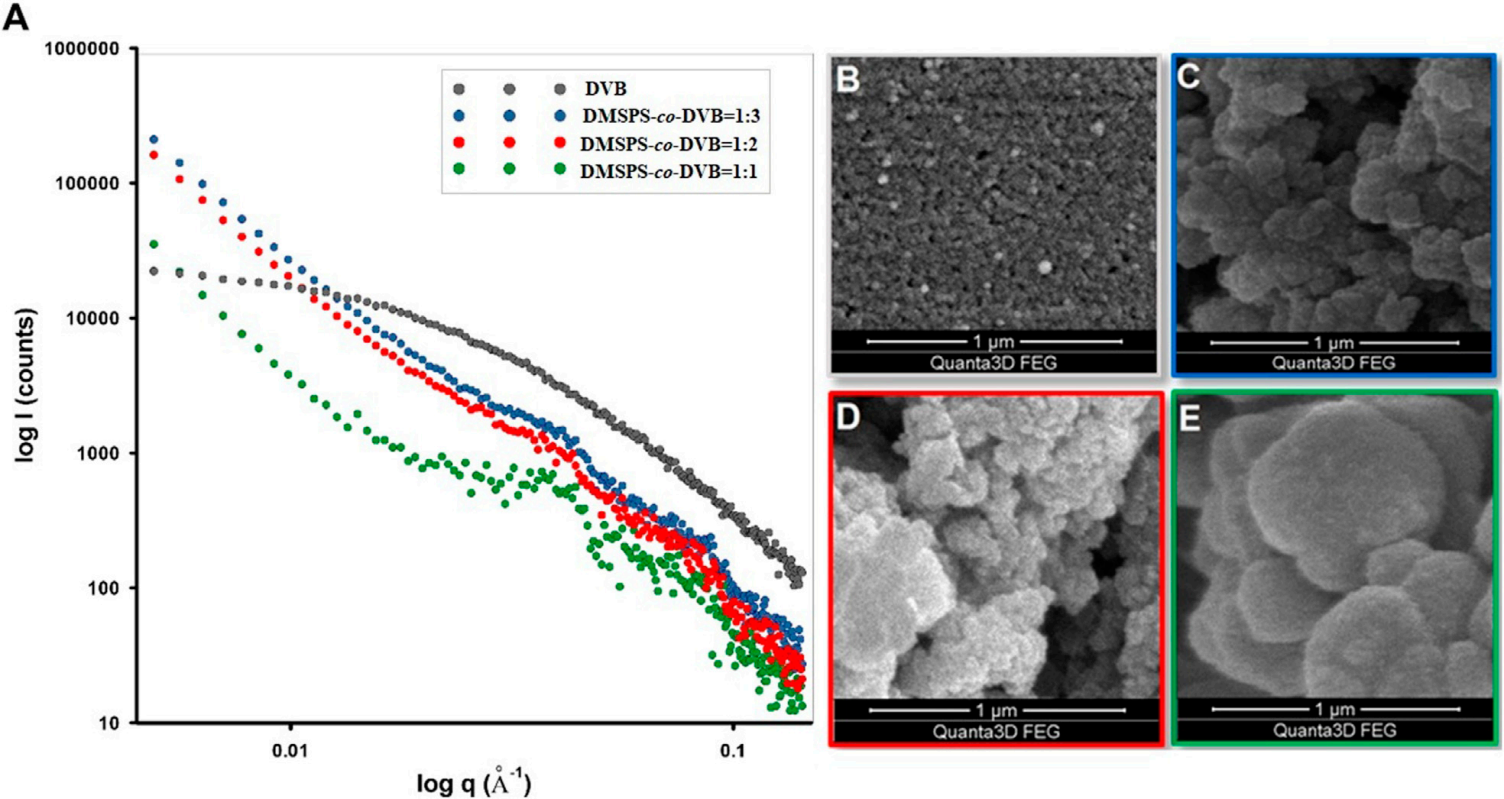
**(A)** Experimental SAXS curves of investigated materials corresponding to the sample morphology presented on SEM images **(B–E)**. The SAXS intensity *versus* momentum transfer *q* (length of the scattering vector) is plotted in the log–log scale.

The observed morphology and bulk properties of nanocomposite materials are directly related to their properties at nanoscale; therefore, the accurate analysis of the nanoscale structure can be a significant step in their characterization. X-ray scattering procedures allow insight into the nanoscale structure by determining many parameters that affect the essential properties. Figure 2 shows the scattering data for the investigated materials.

The intensity versus momentum transfer *q* (length of the scattering vector) is plotted in log–log plots. The SAXS profiles are plain and unstructured for DVB, and fluctuations of scattering intensities for DMSPS-*co*-DVB samples are observed. For the tested samples, there are visible differences in the scattering ability of X-rays in the range of low angles, i.e., in the range of nanometer sizes of scattering objects. It should be noted that the external spheres visible in Figure 1 as structures of approximately 20–100 μm in diameter are too large to be detectable by SAXS within the accessible *q*-range (range of diameters accessible with SAXS measurements is 1–300 nm). Hence, changes in the general course of scattering curves are a direct result of changes in the microstructure and morphology of scattering objects which would be analyzed successively in the later part of the manuscript.

While the SAXS profile is quite homogeneous for DVB, a gradual differentiation of the profile is observed for samples containing DMSPS (DMSPS-*co*-DVB = 1:3, DMSPS-*co*-DVB = 1:2, and DMSPS-*co*-DVB = 1:1). To determine the nature of their origin, spherical systems with a size of 15 nm and zero and non-zero polydispersity were modeled. The results for two selected samples are shown in Supplementary Figure S2. As shown in this figure, the nature of the scattering profile, i.e., the visible fluctuations, can be associated with models of spherical structures with a size of about 15 nm and polydispersity in the range of 10%–15%.

**FIGURE 3:**
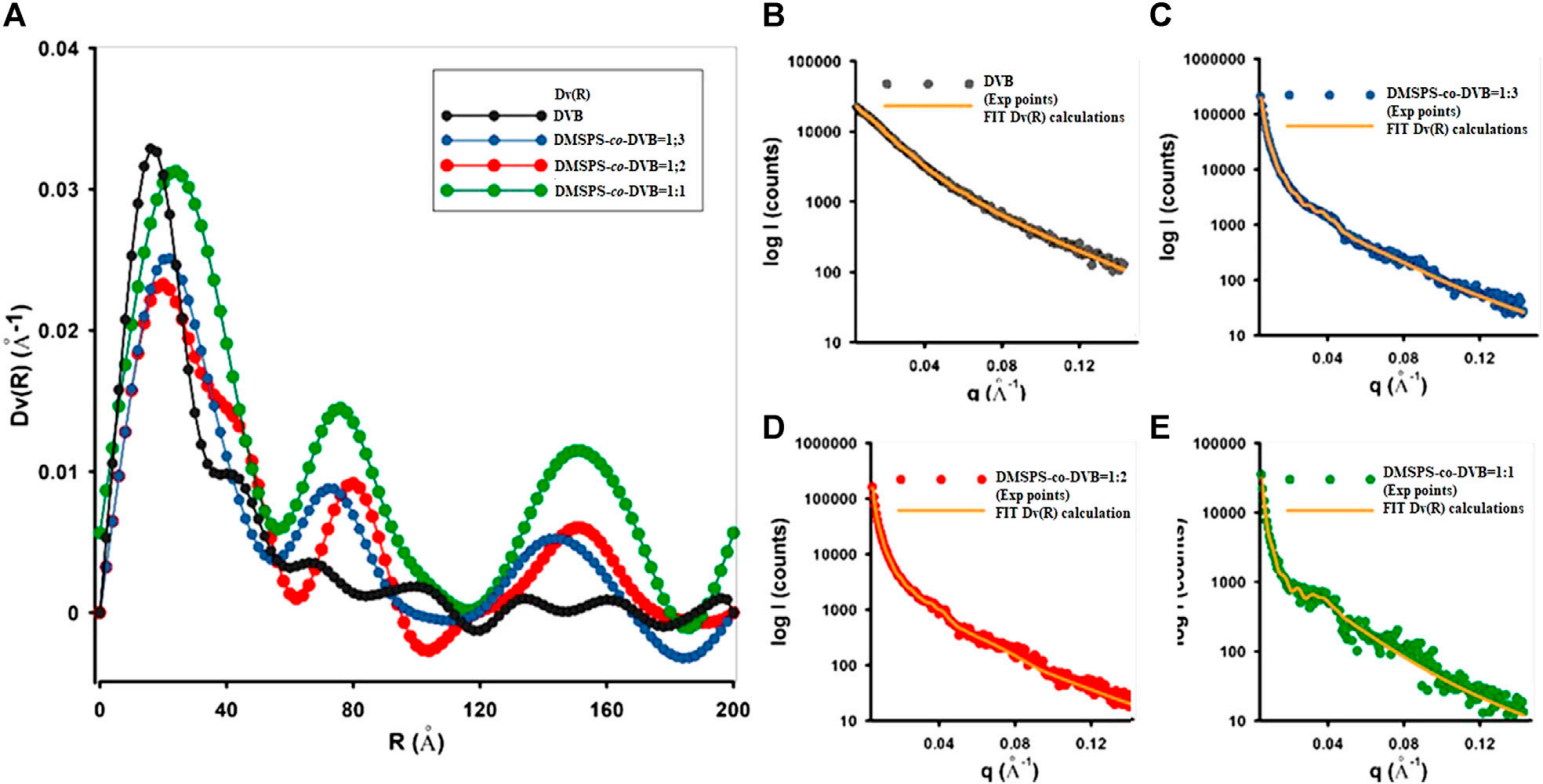
**(A)** Volume size distribution functions *D*_*V*_(*R*) of heterogeneities for the investigated samples and **(B–E)** comparisons of fit curves to the experimental data during *Dv(R)* calculation.

SAXS enables the description of the distribution of scatterers (nanoparticles) in investigated nanocomposites with great statistic volume (typically 1 mm^3^). The size distribution of the scattering objects was analyzed through calculations of the volume-weighted particle size distribution *Dv(R)* function. According to the SAXS theory, the *Dv(R)* algorithm is based on homogeneous, spherical particles without interparticle interactions. Thus, the best fit of the theoretical curve to the experimental data should be expected for probable spherical objects. In this case, the scattering objects with a spherical morphology (satisfying the probable assumption of the spherical shape) have a maximum *Dv(R)* function in the range of 18 Å–55 Å. The exact values are given in Table 2. A reasonable quality of matching the calculations to the experimental data is shown in Figures 3B–E and indicates the correctness of the aforementioned values.

**TABLE 2:** Structural parameters of the investigated systems by SAXS.

Sample name	*Dv(R)* ^a^ (Å)	*PDDF* ^b^ (Å)	*D*_*max*_ (PDDF)^c^ (Å)	Radius of gyration (Å)		Porod approximation				*S*_*SAXS*_^j^ (m^2^/g)
				Guinier analysis^d^	(PDDF)^e^	*K*_*P*_ ^f^	*Q* ^g^ (Å^−1^)	*C*_*0*_ ^h^	*S/V* ^i^ (Å^−1^)	
DMSPS-*co*-DVB = 1:1	35/80/160	600	895	168	170	0.03	2.32	7.75	0.019	80
DMSPS-*co*-DVB = 1:2	22/80/155	490	883	154	166	0.06	7.65	8.21	0.044	220
DMSPS-*co*-DVB = 1:3	21/74/150	400	813	137	123	0.09	10.22	14.14	0.046	230
DVB	20	70	295	86	87	0.30	13.22	24.18	0.092	460

^a^ The *Dv*(*R*) function means the volume-weighted particle size distribution as their maximum value. One or several values are indicated here depending on the number of maxima of the function.

^b^ PDDF means pair distance distribution function as the maximum value of the function for globular geometry.

^c^ Maximum dimension *D*_*max*_ for globular particles. *D*_*max*_ also means R-value (distance) at which PDDF goes to 0. This parameter is defined as the diameter across the longest dimension of the particles and is zero for *r > D*_max_.

^d^ Radius of gyration as the mean-square distance from the center of their distribution. *Rg* provides a measure of the overall size of the scattering objects.

^e^ *Rg* determined from *p(r)* function is proportional to the normalized second moment of *p(r*) from the whole scattering curve.

^f^ Porod constant is proportional to the surface area and the square of the electron density contrast.

^g^ Scattering invariant *Q* is proportional to the mean-square density fluctuation of scattering volume. *Q = 2π* ^*2*^ *·Δρ* ^*2*^ *·V,* where volume *V* and scattering contrast *Δρ*. For the calculation Q invariant, the scattering intensities to *q = 0* and also toward large *q* should be extrapolated.

^h^ Background constant which illustrates asymptotic decay of the SAXS curve at the high *q*.

^i^ Surface-to-volume ratio from the ratio of the Porod constant *K*_*P*_ to the invariant *Q*.

^j^ Surface area by SAXS calculated by $S_{SAXS}=\frac{10000\cdot \frac{S}{V}\left[{Å}^{-1}\right]}{d\;\left[\frac{g}{{cm}^{3}}\right]}$ , where *d* is the density of the material.

Figure 3 shows that divinylbenzene as a linker molecule increases the compactness of the obtained material. Based on the SAXS data, the highest capacity of X-ray scattering among all tested materials and the narrowest size distribution function was noticed for DVB microspheres. The addition of crosslinked copolymers (DMSPS) causes a gradual reduction in scattering capacity (as a result of reducing the number of scattering particles shown in Figure 2) in favor of their increasing size. Moreover, the distribution function becomes more polymodal (DMSPS-*co*-DVB = 1:3 and DMSPS-*co*-DVB = 1:2) until a clear change toward ∼16 nm for DMSPS-*co*-DVB = 1:1. The curves reveal also a gradual increase in the intensity of areas with larger dimensions. Here, three main groups of particle size are visible for the sample DMSPS-*co*-DVB = 1:1.

In conjunction with this data, the Guinier fit provides information on the overall size of the scattering objects. In general, the *R*_*g*_ parameter of this approximation means the root-mean-square distance from the center of density in the molecule. The size of objects with the assumed spherical morphology and the degree of agglomeration of these objects were assessed. Guinier analysis of the SAXS data of investigated samples as the plot of *lnI(q)* vs. *q* ^*2*^ is shown in Supplementary Figure S3 (Supplementary Material).

The DVB sample (Supplementary Figure S3A; Supplementary Material) shows quite good Guinier linearity in the *q*_*max*_ range of 0.0077–0.01507 Å^−1^. This means that the smallest effect of agglomeration should be expected for the sample without DMSPS. For this approximation, the radius of gyration (*R*_*g*_) was calculated as 8.6 nm. An increase in the content of DMSPS in the synthesized product changes not only the linearity range but also the slope of the linear approximation. Supplementary Figures S3B–S5D (Supplementary Material S1) indicate a non-linear trend toward a low scattering vector, where aggregation will be represented by upward experimental points to the Guinier curve. The phenomenon of gradual agglomeration is also shown by increasing values of the square root of the average distance of each scatter from the molecule center. Here, the *R*_*g*_ grows from 13.7 nm to 16.8 nm.

**FIGURE 4:**
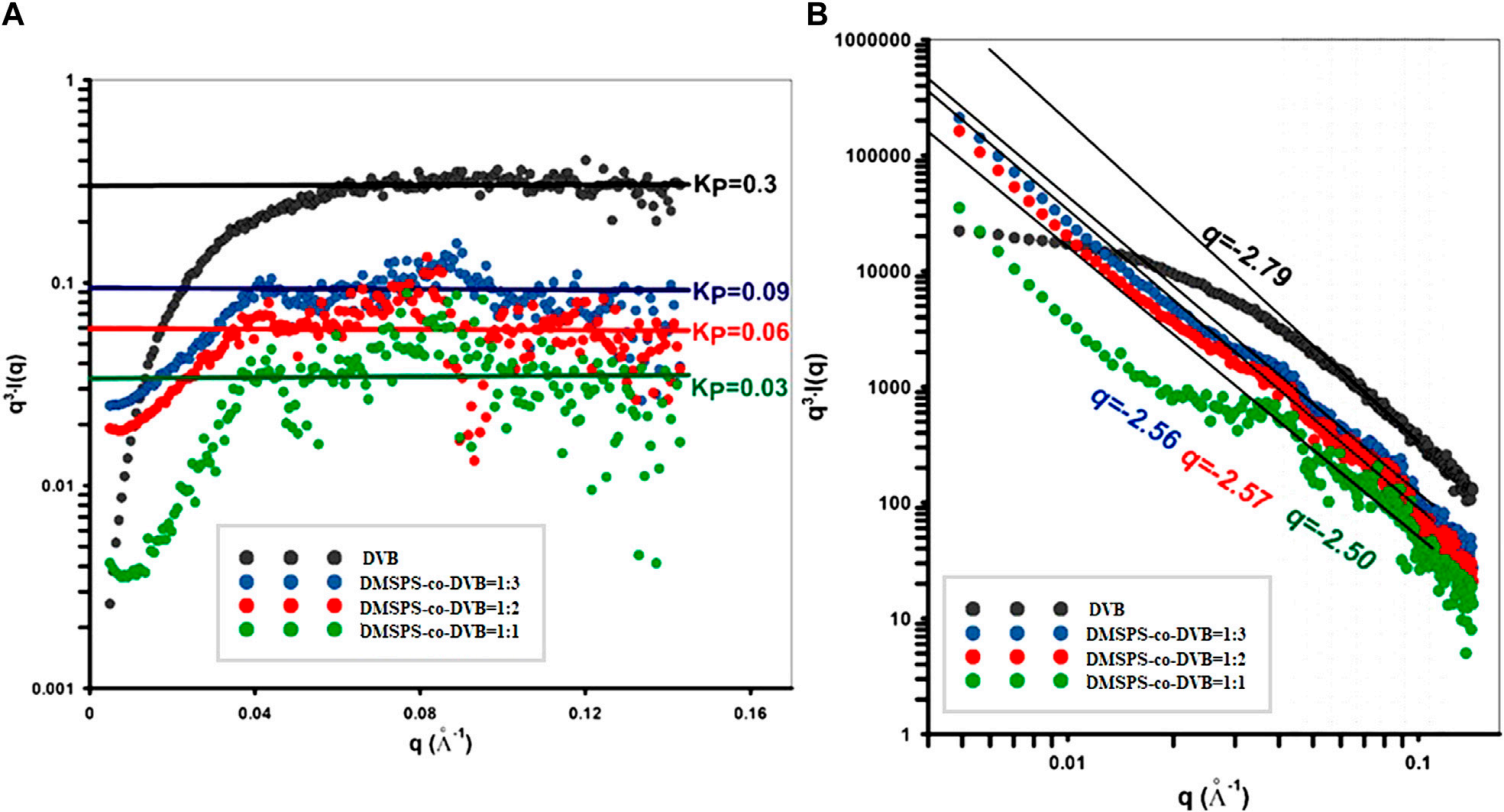
**(A)** Porod fit in order to determine the Porod constant *K*_*P*_ from the intercept of the fit function with the *q*_*3*_ *∙I(q)*. **(B)** Power-law fits of the SAXS data.

The *p(r)* functions (Supplementary Figure S4) show valuable information about the shape and size of particles. The presented calculations allow to complete and confirm the results obtained previously. Pair distance distribution functions of the scattering curves were obtained in the specified range of scattering vector 0.02 < *q* < 0.15. Each synthesized sample was analyzed in terms of PDDF for monodisperse globular particles as a calculation model. The PDDF allows to determine the maximum particle dimension (*D*_*ma*x_) and illustrate the symmetry of spherical particles. Considering the DMSPS-*co*-DVB materials, the spherical structures are described in general with the mean distance of 70 Å, 400 Å, 490 Å, and 600 Å for DVB, DMSPS-*co*-DVB = 1:3, DMSPS-*co*-DVB = 1:2, and DMSPS-*co*-DVB = 1:1 samples, respectively. In these cases, the *p(r*) function goes to zero at *r = 0* and *r = D*_*max*_. However, typically, *p(r)* function should fall gradually to zero at *D*_*max*_ for the samples analyzed in this work; a relatively sharp drop to zero was observed instead of a slight flattening. This means that investigated samples are characterized by underestimated values of *p(r)* functions. The *p(r)* function is forced abruptly down, especially for DMSPS-*co*-DVB = 1:1 sample, where the greatest agglomeration effect was observed. One explanation may be the analysis of electron pair distributions for molecular systems. Particles, macromolecules, and similar systems do not have perfectly sharp boundaries. Therefore, *p(r) = 0*, where the number of electron pairs in the macromolecules/particles are ∼0 at the maximum dimension of the particle (*D*_*max*_). When the agglomeration effect is noticeable such electron density cannot be zero because, for sizes exceeding the size of a particular particle, neighboring structures are present. In addition, the *p(r)* function fits the measured scattering profiles (insets in Supplementary Figures S4A–D; Supplementary Material). Finally, the interfacial surface area and intermediate properties of the investigated materials were considered according to Porod’s theory of scattering data (Figure 4).

The asymptotic behavior of the scattering curves according to Porod’s law was noticed for all samples; however, the characteristic plateau of the Porod function was observed at various levels of *q* ^*3*^ *·I(q)*. The level of the plateau, as indicated by the Porod constant, was the highest for the DVB (*K*_*P*_ = 0.3) sample and successively decreasing for samples in order: DMSPS-*co*-DVB = 1:3, DMSPS-*co*-DVB = 1:2, and DMSPS-*co*-DVB = 1:1. Such a relative comparison of *K*_*P*_ allows determining the relationship between the size of the interfacial surface and the composition of individual samples. It can be expected that the specific surface area (as an interface) will be the largest for the DVB sample and will decrease with increasing the DMSPS content. Moreover, the Porod law is better fulfilled when the interface of the object shows a lower degree of polydispersity (exhibit a smooth surface as for the DVB sample). The power law was compiled within a wide range of the scattering vector according to Porod linear range (gray area in Figure 4B) Here, the exponent parameter was calculated as 2.79, 2.56, 2.57, and 2.50, for DVB, DMSPS-*co*-DVB = 1:3, DMSPS-*co*-DVB = 1:2, and DMSPS-*co*-DVB = 1:1 samples, respectively. Thus, the lower values of this parameter suggest forming of fractal-like structures and scattered heterogeneities in diffuse interface layers.

**FIGURE 5:**
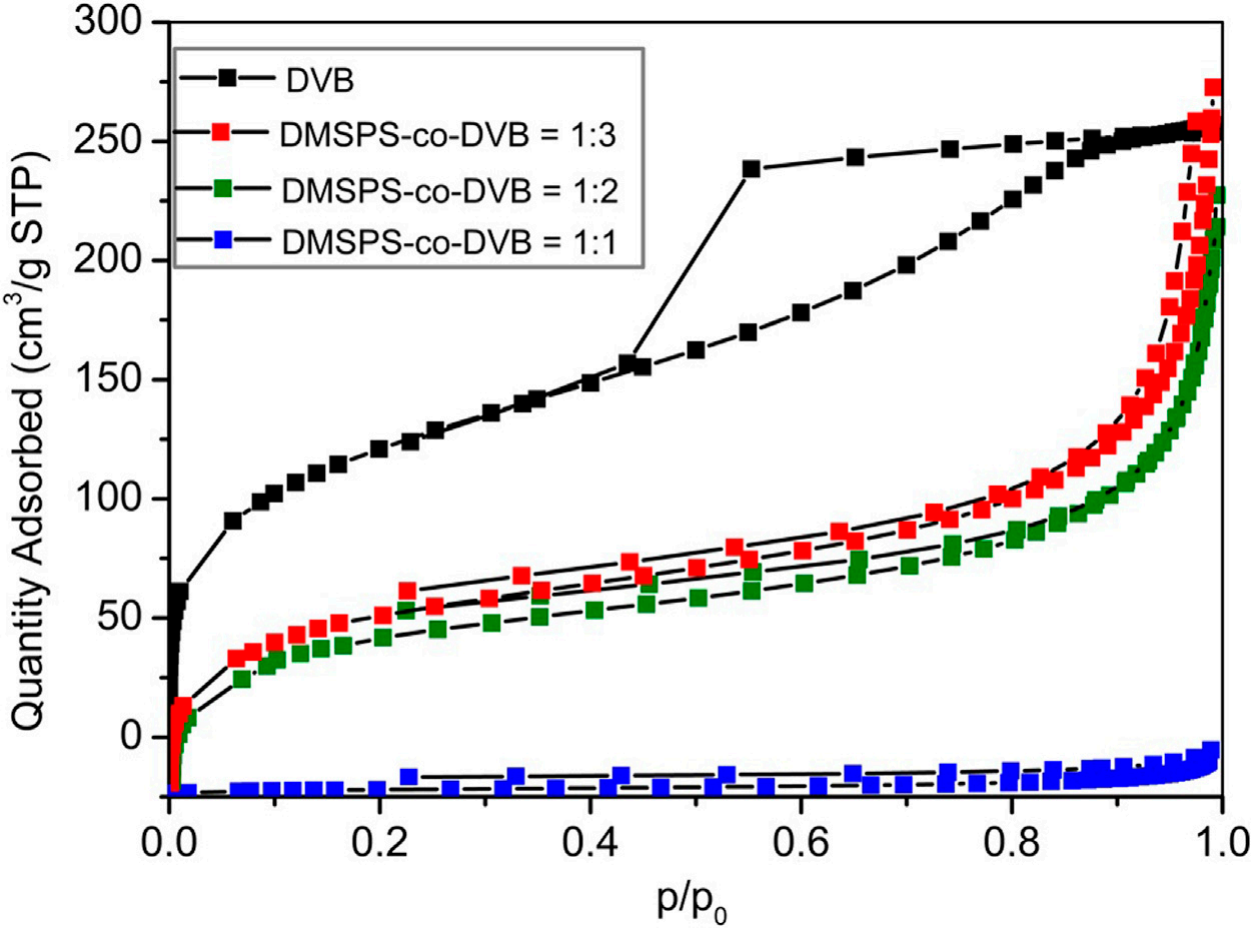
The nitrogen adsorption–desorption isotherms for DMSPS-co-DVB microspheres.

The textural properties of DMSPS-*co*-DVB and DVB microspheres were also investigated by the low-temperature nitrogen adsorption–desorption analysis. In Figure 5, the isotherms for all examined samples are shown. The textural parameters of polymeric materials are presented in Table 3. The differences in the isotherm uptakes are observed. The DVB sample is represented by the IV-isotherm type characteristic for mesoporous materials with hysteresis loop of H2 corresponding to materials with channels of a pore mouth smaller than the pore body (this is the case of ink-bottle-shaped pores) (IUPAC classification). The isotherm shapes of DMSPS-*co*-DVB microspheres are significantly transformed in comparison to DVB (Figure 5), but they can still be classified as the IV-isotherm type typical for mesoporous materials with H3 hysteresis loop, typical for slit-shaped pores (the isotherms revealing H3 type do not show any limiting adsorption at high *p/p*_*0*_, which is observed with aggregates of plate-like particles) (Chang et al., 2009; Alothman, 2012). As can be seen from Table 3, the changes in textural parameters for all polymers are evident. The increase in the values of specific surface area (*S*_*BET*_) and total pore volume (*V*_*t*_) with an increase in the DVB content are observed. The *S*_*BET*_ values for DMSPS-*co*-DVB = 1:1, 1:2, and 1:3 are 9, 225, and 256 m^2^/g, respectively. A similar trend is observed in *V*_*t*_, whose values are 0.02, 0.31, and 0.37 cm^3^/g, respectively. The *S*_*BET*_ and *V*_*t*_ values for DVB are 443 m^2^/g and 0.40 cm^3^/g. The micropore volumes (*V*_*MIC*_) for all tested materials are very low (≤0.01 cm^3^/g). The average hydraulic pore diameters are in the range of 5.4–8.3 nm for DMSPS-*co*-DVB nanomaterials; however, for DVB, the pore size is smaller (3.6 nm). Generally, the determined values are in good compliance with data received from SEM and SAXS investigations.

**TABLE 3:** The textural parameters determined from the nitrogen adsorption–desorption isotherms.

Sample name	*S*_*BET*_ (m^2^/g)	*V*_*t*_ (cm^3^/g)	*V*_*MIC*_ (cm^3^/g)	*D*_*h*_ (nm)	*pH*_*PZC*_
DMSPS-*co*-DVB = 1:1	9	0.02	-	8.3	10.48
DMSPS-*co*-DVB = 1:2	225	0.31	0.01	5.4	10.72
DMSPS-*co*-DVB = 1:3	256	0.37	0.01	5.7	10.85
DVB	443	0.40	0.01	3.6	10.93

The potentiometric titration analysis (Table 2) revealed the alkaline character of the investigated samples (pH_PZC_ > 10). The pH_PZC_ values are 10.48, 10.72, and 10.85 for DMSPS-*co*-DVB = 1:1, 1:2, and 1:3, respectively. The pH_PZC_ value of DVB is 10.93. The decrease in the pH_PZC_ values for DMSPS-*co*-DVB materials in comparison to DVB is related to the content of DMSPS phase. The DMSPS (Supplementary Figure S1; Supplementary Material) is an organic molecule with sulfur atoms and carboxyl groups (a representative of methacrylic thiols) associated with the acidic properties of materials. Overall, the potentiometric titration reveals that the basic/hydrophobic nature of organic molecules predominates in all investigated materials.

**FIGURE 6:**
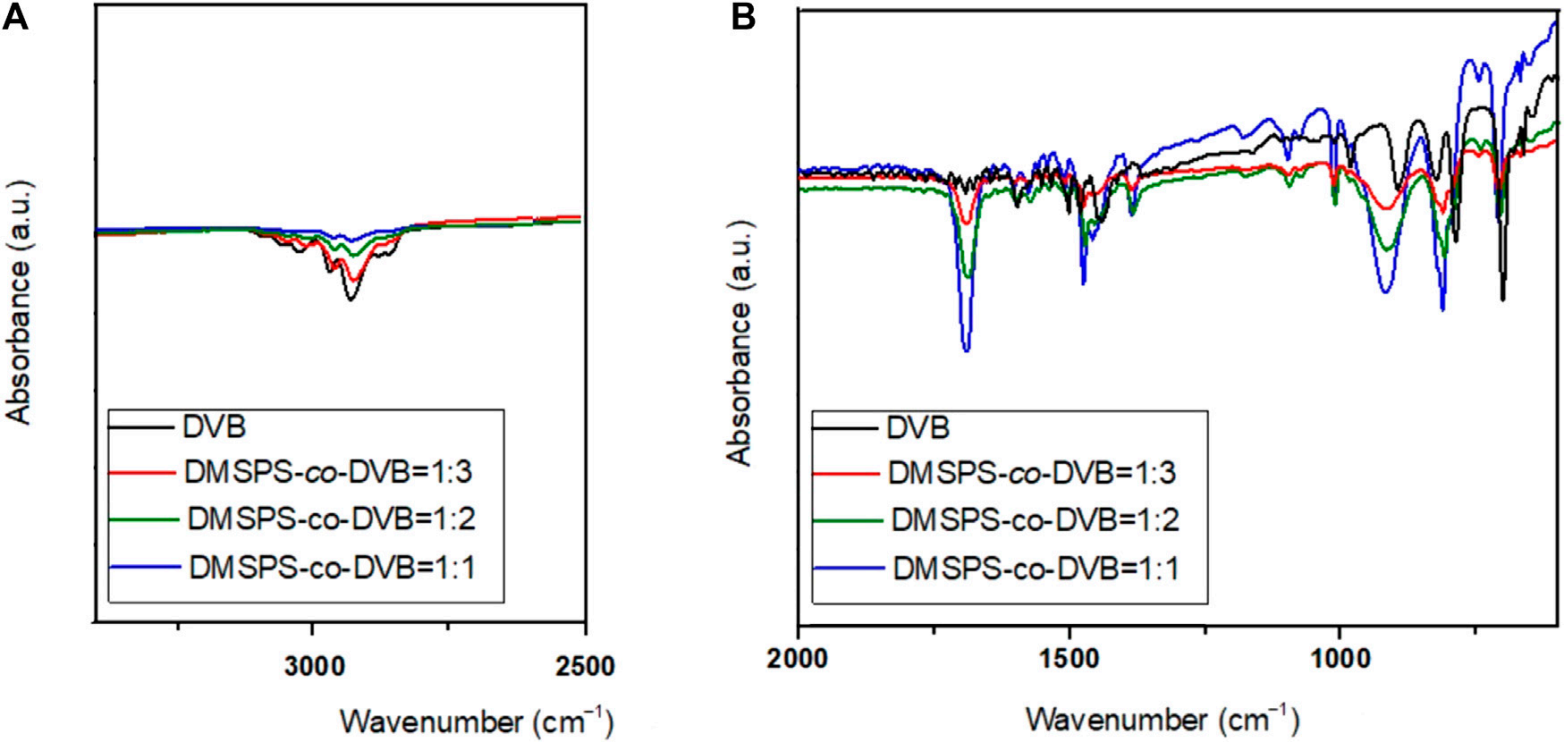
FT-IR/ATR spectra of DMSPS-*co*-DVB and DVB materials in two spectral regions 4000-2500 **(A)**, and 2000-400 **(B)** cm^-1^.

The FT-IR/ATR spectra of DMSPS-*co*-DVB and DVB materials are shown in Figure 6. The FT-IR/ATR technique was used to evaluate the phase bonding efficiency of the DMSPS-*co*-DVB materials. Generally, characteristic vibrations from both phases of DMSPS-*co*-DVB are visible; however, changes in the peak intensity are noted. Both DMSPS and DVB are organic aromatic substances (Supplementary Figure S1; Supplementary Material); therefore, the characteristic vibrations from the benzene ring are visible in the whole spectral range. The stretching vibrations of C–H bonds of the methyl (CH_3_), ethyl (-CH_2_-CH_3_), vinyl (-CH = CH_2_), and aromatic ring groups at 4000–2900 cm^−1^ are reported, but changes in their intensities are related to the composition of the samples (increase in the amount of DMSPS in the DMSPS-*co*-DVB material weakens the intensities of the peaks in this spectral region, which confirms the correctness of DMSPS binding with DVB). The stretching vibrations of the carboxyl group (C=O) at ∼1700 cm^−1^ and C=C at 1650 cm^−1^ are also observed. The stretching vibrations of C-O-C are detected for DMSPS-*co*-DVB materials in the range of 1200–1100 cm^−1^, which are invisible to DVB (confirmation of the synthesis correctness). The peaks from aromatic ring stretches are seen at 1600, 1500, and 1450 cm^−1^. The deformation vibrations of methyl and ethyl groups are observed in the range of 1470–1350 cm^−1^. The deformation bands of C-H groups of the benzene ring in the range of 1000–650 cm^−1^ are also observed. Overall, the FT-IR/ATR analysis confirmed successful phase bonding for biphasic polymeric systems. The DMSPS-co-DVB systems result in the presence of more C-O-C and C=O groups compared to DVB which results from the methacrylic nature of the DMSPS phase. Generally, the FT-IR analysis confirms the strong organic aromatic (hydrophobic) character of the investigated samples. The data obtained from potentiometric titration and FT-IR spectroscopy are in good compatibility (Bosacka et al., 2021).

The series of DMSPS-*co*-DVB nanocomposites were tested in terms of their sorption characteristics. For this purpose, the adsorption kinetic studies for aniline on the obtained materials as adsorbents were conducted. The selection of this organic compound as a potential pollutant was dictated by the fact that it is widely used in various branches of the industry, i.e., pharmaceutical, rubber, dyeing, agrochemical, and synthetic. The last reports show that aniline has been detected in municipal wastewater, surface water, and even in groundwater. According to the Environmental Protection Agency (EPA), aniline is a probable carcinogen and it has been classified as Group B2. Therefore, in the environmental aspect, the development of efficient methods for aniline removal from water is of great importance (Gao et al., 2015; Ahmadi and Igwegbe, 2018; Shang et al., 2020; Gu et al., 2021).

**FIGURE 7:**
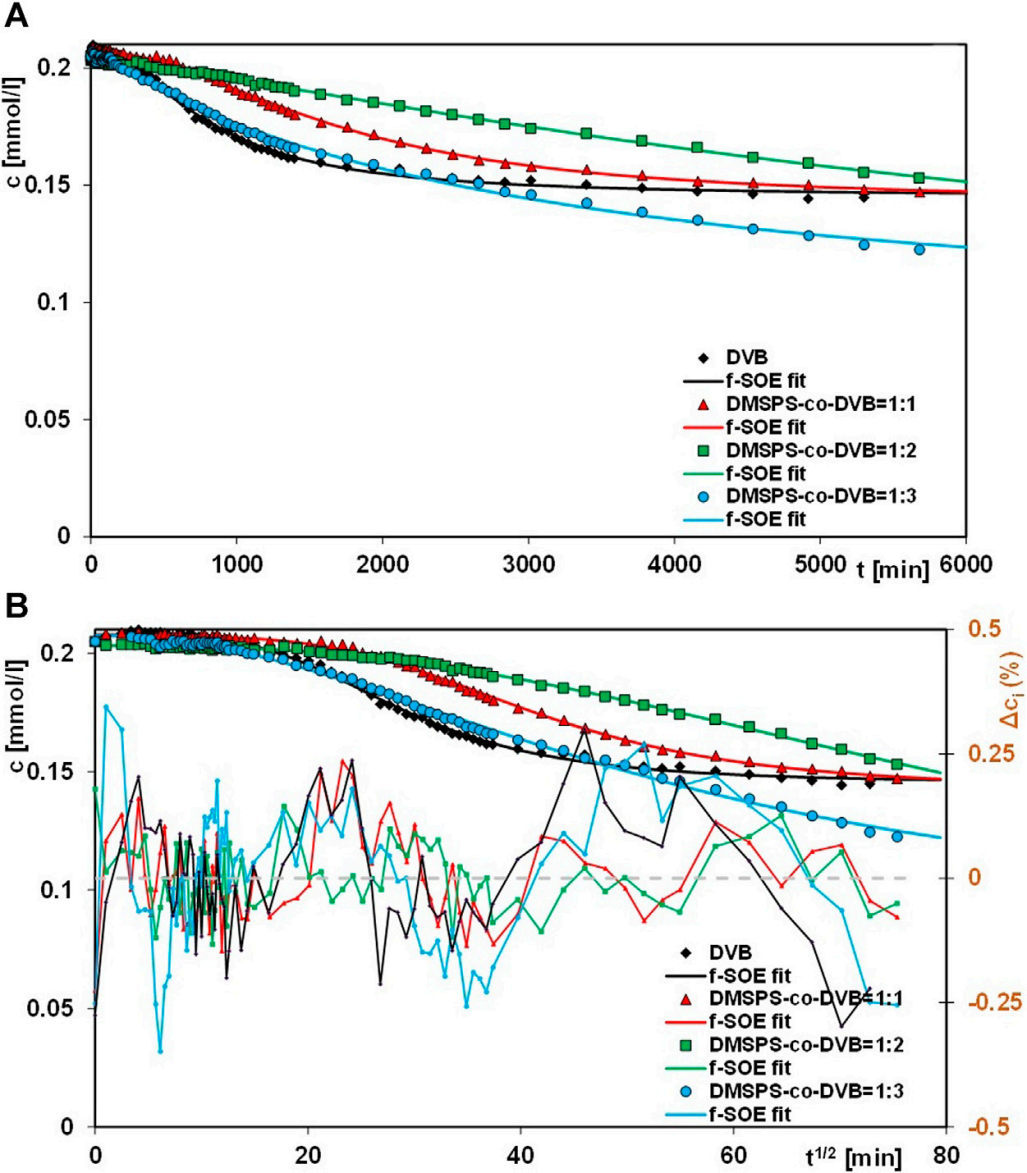
Comparison of adsorption kinetics for aniline on the DVB and DMSPS-*co*-DVB nanocomposites at the coordinates: concentration ∼ time **(A)** and concentration ∼ square root of time **(B)**. The lines correspond to the fitted f-SOE equation.

**FIGURE 8:**
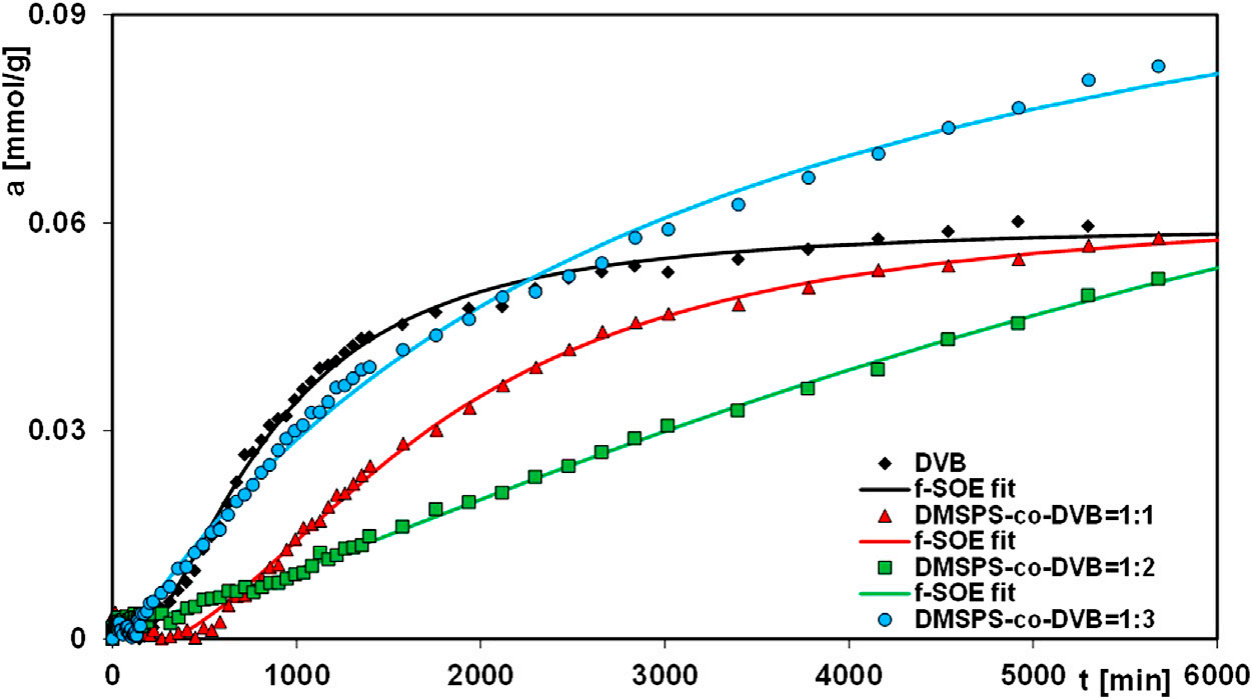
Comparison of adsorption kinetics for aniline on the DVB and DMSPS-*co*-DVB nanocomposites at the adsorption ∼ time coordinates. The lines correspond to the fitted f-SOE equation.

**FIGURE 9:**
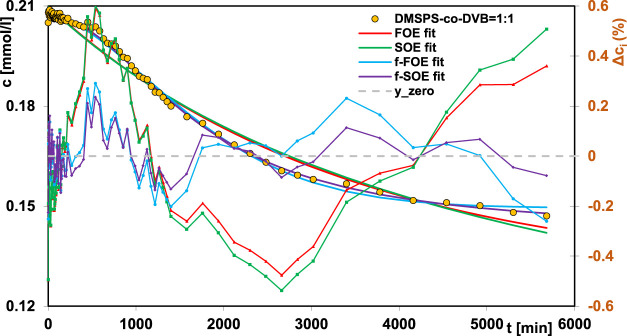
Comparison of the adsorption kinetics for aniline on the DMSPS-*co*-DVB = 1:1 nanocomposite fitted to the FOE, SOE, f-FOE, and f-SOE equations.

In this paper, the results of aniline elimination from water by the adsorption technique applying novel polymeric nanocomposites are presented. For comparative purposes, the adsorption experiments were extended to the DVB material. Figures 7–9 show the profiles of changes in concentration and adsorption over time for the studied systems. One can see the differentiation in the adsorption rate and the pollutant adsorbed amounts on respective nanocomposites in the course of the experiments. These observations can be explained by differences in morphological and structural characteristics of the materials, whose synthesis procedures assumed variable ratios of DMSPS and DVB in the reaction mixtures. The aniline adsorption process was the fastest and most efficient for the DMSPS-*co*-DVB = 1:3 material. Undoubtedly, the process was favored by the porous structure of the solid formed by agglomeration of particles with spherical morphology (well-developed specific surface area and high total pore volume). On the contrary, adsorption active sites in the forms of benzene rings and thiol groups were provided by the DVB and DMSPS components, respectively.

The kinetic curves showing aniline adsorption on DMSPS-*co*-DVB = 1:1 (1) and DMSPS-*co*-DVB = 1:2 (2) nanomaterials intersect in the initial period and are getting closer to each other at the final time of the experiment. To explain the observed phenomena, two factors were considered: the type and number of nanomaterial active sites and their accessibility to the pollutant. The DMSPS-*co*-DVB = 1:1 polymer is characterized by the extensive fractal structure revealed on the SEM images in Figures 1J–L and confirmed by the value of the exponent parameter *q* determined from Porod’s law for the SAXS data (Figure 7B). This type of structure was responsible for a greater rate of aniline adsorption on the DMSPS-*co*-DVB = 1:1 than on DMSPS-*co*-DVB = 1:2 up to about 2800 min of the experiment. Furthermore, the process slowed down and eventually became slower than that on DMSPS-*co*-DVB = 1:2. The reason for it can be found in the decrease in number of benzene rings related with the amount of the DVB component.

The mechanism of the aniline adsorption on the DMSPS-*co*-DVB nanocomposites was mainly based on π–π interactions between adsorbate benzene rings and the aromatic parts of the adsorbent. This thesis may be confirmed by a course of the adsorption curves in a time function for the investigated systems, as shown in Figure 8. At the final time of the experiment (6000 min), the polymer with the highest contribution of the aromatic component DVB (DMSPS-*co*-DVB = 1:3) is characterized by the greatest adsorption equal to 0.083 mmol/g. The aniline adsorption amounts for DMSPS-*co*-DVB = 1:1 and DMSPS-*co*-DVB = 1:2 are 0.057 and 0.052 mmol/g, respectively. However, the adsorption level for the two last nanopolymers should be evaluated after extending of the experiment time close to the adsorption equilibrium state due to the significant differences in slope of adsorption curves after 6000 min. Based on this, it can be expected that the maximum aniline adsorption value for DMSPS-*co*-DVB = 1:2 will exceed adsorption in the system with DMSPS-*co*-DVB = 1:1.

One can observe that the aniline adsorption kinetics on the DVB is faster than on DMSPS-*co*-DVB nanopolymers. After 4000 min, a state close to adsorption equilibrium was already achieved. The maximum aniline adsorption value on DVB equals 0.059 mmol/g and is near to those, obtained for DVB = 1:1 and DMSPS-*co*-DVB = 1:2 materials (0.057 and 0.052 mmol/g). However, the efficiency of the adsorption process on DMSPS-*co*-DVB = 1:3 is the greatest (0.083 mmol/g). The superiority of DMSPS-*co*-DVB = 1:3 as an adsorbent over DVB results from morphological, structural, and textural features. Both samples are in the form of microspheres, but for the biphasic nanopolymers, a higher tendency to form the agglomerates (due to the presence of the DMSPS component) was reported. Also, the surface of DMSPS-*co*-DVB nanopolymers is rougher in comparison to DVB. In turn, DVB microspheres are more compact, with a smooth surface and much lower agglomeration effect. Texturally, both materials are characterized by a relatively well-developed specific surface area (256 and 443 m^2^/g for the DMSPS-*co*-DVB = 1:3 nanomaterial and DVB, respectively) and a significant total pore volume (0.37 and 0.40 cm^3^/g for the DMSPS-*co*-DVB = 1:3 nanopolymer and DVB, respectively). However, they have different types of pore shapes (i.e., slit-shaped and ink-bottle-shaped pores, with average pore diameters of 5.7 and 3.6 nm, for the DMSPS-*co*-DVB = 1:3 nanomaterial and DVB, respectively). Taking all this into account, it can be assumed that the co-polymerization process used in the synthesis of DMSPS-*co*-DVB = 1:3 nanopolymer caused a formation of a solid with a less compact structure than the DVB nanomaterial, which allowed to increase the interfacial surface for better contact of the adsorbate with adsorbent favoring aniline adsorption. An increase in pore size and an alteration in pore shape (narrow entries to inter- and intra-particle pores in DVB and slit-shaped pores in the DMSPS-*co*-DVB = 1:3 nanopolymer) affected the adsorption process.

The aniline maximum adsorption values on DMSPS-*co*-DVB nanopolymers are compared with other materials presented in the literature (Table 4). The obtained results are better than for many materials produced from raw substances, e.g., Kraft lignin, pine sawdust, almond, or for MWCNTs/ferrite nanocomposite, and are comparable to some clays, e.g., modified montmorillonite or zeolites.

**TABLE 4:** Comparison of the selected literature data of aniline maximum adsorption values on different types of materials with DMSPS-*co*-DVB nanopolymers.

No.	Sample name	*a*_*m*_ (mmol/g)	Literature
1	DMSPS-*co*-DVB = 1:3 nanopolymer	0.083	-
2	Pine sawdust (PS)	0.016	Zhou et al. (2014)
3	MWCNTs/ferrite nanocomposite	0.022	Salam et al. (2012)
4	Prunus dulcis (almond)	0.035	Rahdar et al. (2017)
5	Kraft lignin	0.071	Jiang et al. (2019)
6	Modified montmorillonite Clay	0.086	Dulail and Elbasheer (2023)
7	ZSM-5 zeolite	0.089	Abbas and Hussien (2017)

The experimental kinetic profiles were analyzed using the following theoretical models: the FOE, the SOE, and the fractal-like MOE/the pseudo fractal-like MOE (f-MOE/f-PMOE) equation. Optimization with the last equation for each adsorption system gave the values of the parameter *f*_*2*_ equal 0 or 1, so f-MOE (for *f*_*2*_ ∈ <0,1>) was reduced to f-FOE or f-SOE. In Tables 5, 6, the comparison of the parameters calculated from the theoretical approaches and the values of relative deviation *SD(c)/c*_*o*_ are collected. For two DMSPS-*co*-DVB = 1:2 and DMSPS-*co*-DVB = 1:3 nanomaterials, a very good correlation between the experimental data and applied kinetic models was obtained. In turn, the aniline adsorption process on the DMSPS-*co*-DVB = 1:1 nanopolymer was successfully described by the f-FOE and f-SOE equations, and the other equations gave worse results, suggesting heterogeneity of this system. The obtained values of the fractal parameter *p*, i.e., 1.63 and 2 (from f-FOE and f-SOE, respectively), confirmed the validity of the observations. In Figure 9, the experimental data of DMSPS-*co*-DVB = 1:1 material, fitted with the FOE, SOE, f-FOE, and f-SOE equations, as well as their deviations, are plotted.

**TABLE 5:** Comparison of the parameters of various kinetic equations.

Sample name	fit	*f*_*2*_ */p*	*log k* ^*^	*t*_*0.5*_ (min)	*u*_*exp_f*_	*u*_*eq*_	*1-R* ^*2*^
DMSPS-*co*-DVB = 1:1	FOE	0	−3.53	2,329	0.317	0.388	1.85 · 10^−2^
	SOE	1	−3.72	5,253	0.324	0.623	2.11 · 10^−2^
	f-FOE	0/1.63	−3.29	1,564	0.279	0.280	3.53 · 10^−3^
	f-SOE	1/2	−3.23	1,714	0.287	0.313	2.15 · 10^−3^
DMSPS-*co*-DVB =1:2	FOE	0	−4.30	13,828	0.248	1.000	5.11 · 10^−3^
	SOE	1	−4.26	18,034	0.239	1.000	8.80 · 10^−3^
	f-FOE	0/1.26	−3.77	4,362	0.244	0.399	2.78 · 10^−3^
	f-SOE	1/1.30	−3.87	7,485	0.244	0.598	2.72 · 10^−3^
DMSPS-*co*-DVB = 1:3	FOE	0	−3.38	1,647	0.390	0.429	4.83 · 10^−3^
	SOE	1	−3.49	3,059	0.399	0.615	3.80 · 10^−3^
	f-FOE	0/0.91	−3.45	1,878	0.399	0.462	4.37 · 10^−3^
	f-SOE	1/1.03	−3.45	2,831	0.398	0.586	3.76 · 10^−3^
DVB	FOE	0	−3.06	782	0.314	0.316	1.08 · 10^−2^
	SOE	1	−3.09	1,223	0.335	0.413	1.72 · 10^−2^
	f-FOE	0/1.40	−3.01	779	0.286	0.286	5.15 · 10^−3^
	f-SOE	1/1.91	−2.92	824	0.293	0.301	2.81 · 10^−3^

k^*^: k_1_-FOE, f-FOE, and MOE; k_2_-SOE and f-SOE.

**TABLE 6:** Comparison of the relative deviation SD(c)/c_o_ values for various kinetic equations.

Sample name	FOE (%)	SOE (%)	f-FOE (%)	f-SOE (%)
DMSPS-*co*-DVB =1:1	1.26	1.35	0.56	0.44
DMSPS-*co*-DVB =1:2	0.44	0.58	0.33	0.33
DMSPS-*co*-DVB = 1:3	0.83	0.74	0.80	0.74
DVB	1.16	1.46	0.81	0.60
Average	0.92	1.03	0.63	0.53

## 3 Conclusion

In this work, the S,S′-thiodi-4,1-phenylene bis(thio-methacrylate)-*co*-divinylbenzene (DMSPS-*co*-DVB) nanopolymers were successfully synthesized in a polymerization reaction using different proportions of monomers: 1:1, 1:2, and 1:3. The morphological, structural, and textural properties of the obtained nanomaterials were investigated using SEM, SAXS, and nitrogen sorption. Generally, the textural/morphological characteristics of biphasic systems based on DMSPS and DVB were different (non-ideal spherical shape of grains, higher tendency to form agglomerates, and roughness of surface) compared to DVB (spherical shape of grains, smoothness of surface, and lower agglomeration) and varied with the synthesis mixture composition. The SAXS analysis allowed analyzing the nanopolymer structure in detail and confirmed the data obtained from SEM. The results based on nitrogen sorption analysis were also in good agreement with the SEM and SAXS data. The potentiometric titration and FT-IR/ATR analysis revealed mostly the alkaline/hydrophobic character of investigated systems (related to the aromatic structures of both DMSPS and DVB molecules); however, the certain influence of thiol nature of DMSPS was evident. The kinetic studies of aniline on the DMSPS-*co*-DVB nanomaterials revealed the global effect of factors that may influence the adsorption process. One of the important factors distinguishing the systems based on DMSPS and DVB was the tendency of individual polymer spheres to clump together, which is the reason why they were exceptionally well described by the fractal model of adsorption kinetics. One can find that the synthesized materials show noticeable selectivity toward aniline. The presented studies showed that depending on the synthesis procedure, it is possible to receive the materials which are characterized by divergent uptakes and kinetic characteristics.

## Data Availability

The raw data supporting the conclusion of this article will be made available by the authors, without undue reservation.
