# Supplementary material for: Physicochemical, structural, and adsorption characteristics of DMSPS-co-DVB nanopolymers
Source: Front Chem. 2023 Jun 28;11:1176718. doi: 10.3389/fchem.2023.1176718 (PMC10338118; doi:10.3389/fchem.2023.1176718)
Supplement: Supplementary file 1 [file DataSheet1.docx]

Supplementary Material

Physicochemical, Structural and Adsorption Characteristics of DMSPS-*co*-DVB Nanopolymers

Alicja Bosacka^1,2,*^, Malgorzata Zienkiewicz-Strzalka^2^, Anna Derylo-Marczewska^2^, Agnieszka Chrzanowska^2^, Magdalena Blachnio^2^, Beata Podkoscielna^3^

^1^ Department of Fundamental Technologies, Faculty of Production Engineering, University of Life Sciences, Gleboka Street 28, 60-612, Lublin, Poland

^2^ Department of Physical Chemistry, Faculty of Chemistry, Institute of Chemical Sciences, Maria Curie-Sklodowska University, Maria Curie-Sklodowska Sq. 3, 20-031, Lublin, Poland

^3^ Department of Polymer Chemistry, Faculty of Chemistry, Institute of Chemical Sciences, Maria Curie Skłodowska University, Gliniana 33, 20-614 Lublin, Poland

*** Correspondence:**alicja.bosacka@up.lublin.pl

Keywords: polymers, microspheres, surface properties, aniline adsorption, nanomaterials

| **A**    **DMSPS** | **B**    **DVB** |
| --- | --- |

**Figure S1.** Chemical structures of monomers: S,S'-thiodi-4,1-phenylene bis(thiomethacrylate) (DMSPS) (A) and divinylbenzene (DVB) (B).

**Table S1.** Aniline physicochemical characteristics^1^.

| Compound name | Aniline |
| --- | --- |
| Molecule structure | C_6_H_7_N |
| Chemical formula |  |
| Molar mass (g/mol) | 93.13 |
| Density(g/mL) | 1.02 |
| Solubility in water at 20 °C (g/100 mL) | 3.6 |
| pKa | 4.63 |
| Melting point (°C) | −6.3 |
| Boiling point (°C) | 184.1 |

^1^https://pubchem.ncbi.nlm.nih.gov(Access date: 01.02.2023)

**
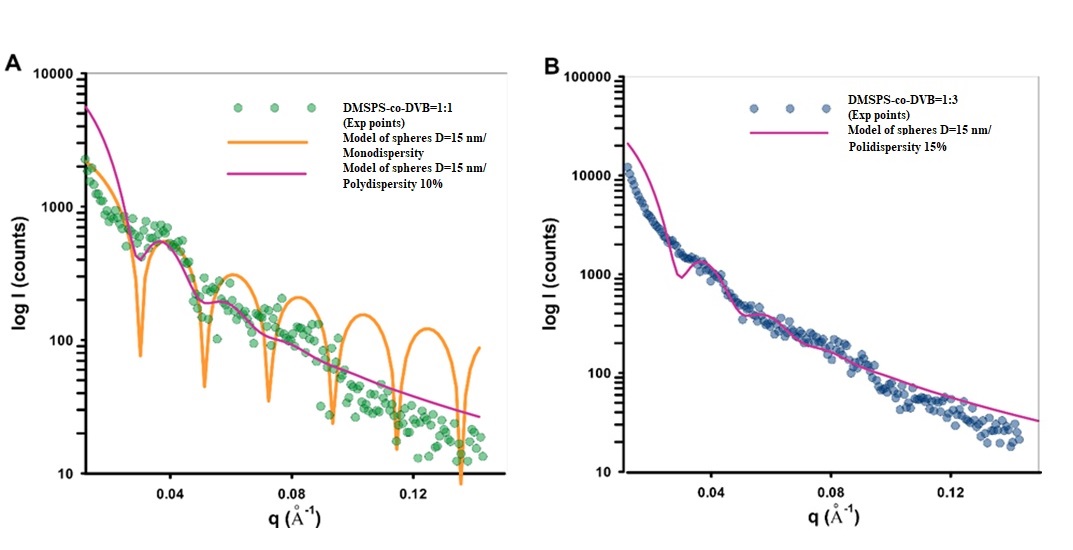
**

**Figure S2.** Simulated SAXS curves for spherical particles varying the degree of polydispersity for two selected samples: (A) DMSPS-*co*-DVB=1:1 and (B) DMSPS-*co*-DVB=1:3 and its fitting to experimental points.


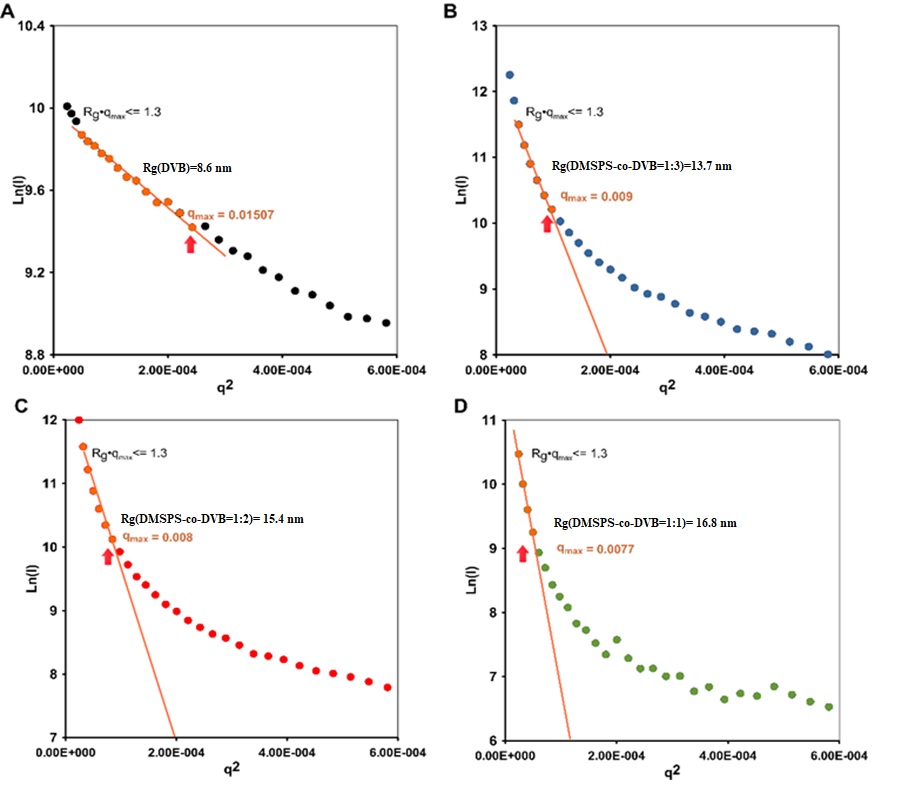


**Figure S3.**Guinier analysis of the SAXS data of investigated samples as the plot of ln I(q) vs q^2^ from the Guinier approximation: (A) DVB, (B) DMSPS-*co*-DVB=1:3, (C) DMSPS-*co*-DVB=1:2, and (D) DMSPS-*co*-DVB=1:1. The maximum extent of the Guinier region defined as *Rg·q* values was achieved as ≤1.3. These values were chosen to have <10% error resulting from the deviation of the spherical shape from the Guinier approximation.


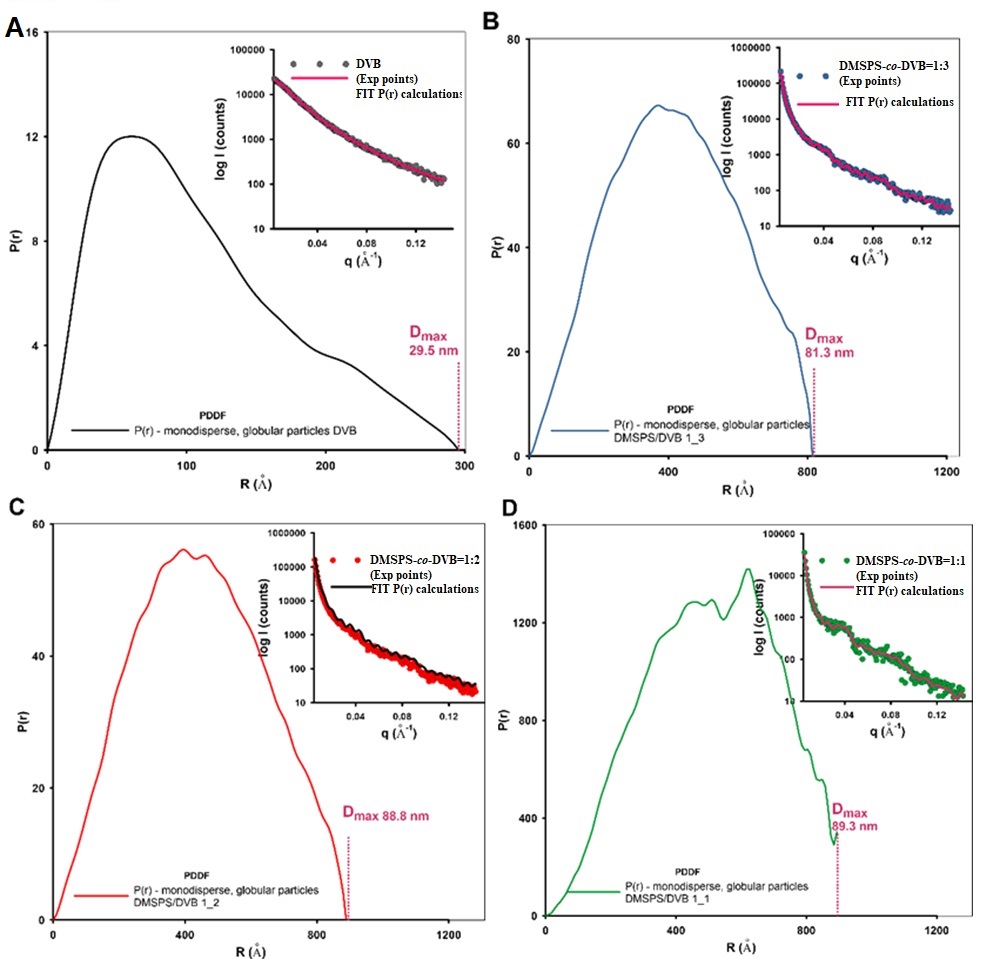


**Figure S4.** The pair distance distribution functions of investigated samples *p(r)* calculated from the SAXS scattering profiles for DVB, DMSPS-*co*-DVB=1:3, DMSPS-*co*-DVB=1:2 and DMSPS-*co*-DVB=1:1 and corresponding Fit curves of *P(r)* calculations vs. experimental points.
